# The analysis of egg and milk allergens in children patients

**DOI:** 10.1186/2045-7022-1-S1-P21

**Published:** 2011-08-12

**Authors:** Li He, Qin Yuan Chen

**Affiliations:** 1 Women and Children Health Institute of Futian, Shenzhen, China, Pediatrics, Shenzhen, China

## Background

To investigate the Status of children allergic toegg and milk.

## Methods

Serum special IgE antibody (SIgE) levels against egg and milk allergens were detected by fluorescent ELISA.

## Results

The positive rates of SIgE against the egg allergen and the milk allergen of children were 33.1% and 25.7%, respectively. The positive rates of egg allergen in 0-3, 3-6 years group were significantly higher than those of 6-14 years group(P< 0.05). The positive rates of milk allergen in 0-3 years group were significantly higher than those of 3-6,6-14 years group (P<0.05).

## Conclusions

The positive rates of egg and milk allergens in children were decrease as the age of children increased.

## Key words

Egg, Milk, SIgE, Food allergy, Children

